# Capsaicin, The Vanilloid Receptor TRPV1 Agonist in Neuroprotection: Mechanisms Involved and Significance

**DOI:** 10.1007/s11064-023-03983-z

**Published:** 2023-07-26

**Authors:** Omar M.E. Abdel-Salam, Gyula Mózsik

**Affiliations:** 1 Department of Toxicology and Narcotics, Medical Research and Clinical Studies Institute, National Research Centre, Cairo, Egypt; 2 First Department of Medicine, Medical and Health Centre, University of Pécs, H-9724 Pecs, Hungary

**Keywords:** Sensory neurons, Capsaicin, Resiniferatoxin, *Capsicum*, Hot peppers, TRPV1, Brain stroke, Systemic inflammation, Neurodegenerative diseases, Epilepsy

## Abstract

Hot peppers, also called chilli, chilli pepper, or paprika of the plant genus *Capsicum* (family *Solanaceae*), are one of the most used vegetables and spices worldwide. Capsaicin (8-methyl N-vanillyl-6-noneamide) is the main pungent principle of hot green and red peppers. By acting on the capsaicin receptor or transient receptor potential cation channel vanilloid subfamily member 1 (TRPV1), capsaicin selectively stimulates and in high doses defunctionalizes capsaicin-sensitive chemonociceptors with C and Aδ afferent fibers. This channel, which is involved in a wide range of neuronal processes, is expressed in peripheral and central branches of capsaicin-sensitive nociceptive neurons, sensory ganglia, the spinal cord, and different brain regions in neuronal cell bodies, dendrites, astrocytes, and pericytes. Several experimental and clinical studies provided evidence that capsaicin protected against ischaemic or excitotoxic cerebral neuronal injury and may lower the risk of cerebral stroke. By preventing neuronal death, memory impairment and inhibiting the amyloidogenic process, capsaicin may also be beneficial in neurodegenerative disorders such as Parkinson’s or Alzheimer’s diseases. Capsaicin given in systemic inflammation/sepsis exerted beneficial antioxidant and anti-inflammatory effects while defunctionalization of capsaicin-sensitive vagal afferents has been demonstrated to increase brain oxidative stress. Capsaicin may act in the periphery *via* the vagal sensory fibers expressing TRPV1 receptors to reduce immune oxidative and inflammatory signalling to the brain. Capsaicin given in small doses has also been reported to inhibit the experimentally-induced epileptic seizures. The aim of this review is to provide a concise account on the most recent findings related to this topic. We attempted to delineate such mechanisms by which capsaicin exerts its neuronal protective effects. We also aimed to provide the reader with the current knowledge on the mechanism of action of capsaicin on sensory receptors.

## Introduction

Capsaicin (8-methyl-*N*-vanillyl-6-nonenamide) is the main pungent principle in hot chilli peppers of the plant genus *Capsicum*, a member of the *Solanaceae* family. The species most commonly grown around the world is *Capsicum annuum*. *Capsicum* contains up to 1.5% pungent principles, commonly composed of capsaicin, dihydrocapsaicin, and others. The content of capsaicin in hot peppers varies between 0.1 and 1.2%. The second main capsaicinoid in hot peppers is dihydrocapsaicin [1, 2]. The majority of individuals, if not all of them, are familiar with the hot burning sensation perceived when the *Capsicum* fruit is introduced into the oral cavity. This pungent flavor or the sensory experience of eating hot peppers may account for their inclusion in spicy food recipes and a variety of restaurant cuisines, making *Capsicum* the most consumed spice in the world [3].

The most important function of pungent capsaicin, however, is that it is a lead molecule in sensory pharmacology, a chemical probe for a subset of primary sensory neurons and their nerve fibers [4]. Capsaicin has this neuroselective site of action, the capsaicin-sensitive nociceptor neurons. These neurons are of small to medium diameter, and give rise to unmyelinated (C fiber) and thinly myelinated axons (Aδ fibers). These fibers transmit nociceptive information arising from the viscera and skin to the central nervous system while also releasing their peptide content in the periphery (efferent function) which has an impact on numerous local tissue processes [5, 6, 7]. These nociceptive sensory neurons have cell bodies in the spinal and cranial sensory ganglia, peripheral axons that innervate the skin and peripheral tissues, and central branches that enter the spinal cord *via* the dorsal roots, synapse with second order neurons to convey information to the central nervous system [7, 8].

The molecular site of action for capsaicin is the capsaicin receptor, first proposed by Szolcsányi in 1975 [9] or the transient receptor potential cation channel vanilloid subfamily member 1 (TRPV1) [10] which is expressed by polymodal capsaicin-sensitive nociceptive neurons in their central branches and peripheral terminals and also in trigeminal ganglion neurons, vagal afferents in jugular and nodose ganglion neurons [8, 11, 12]. In the central nervous system, TRPV1 channel receptors have been detected in the spinal cord and in the brain where they are expressed in neuronal cell bodies and dendrites, as well as in non-neuronal cells e.g., astrocytes, and pericytes in several brain regions e.g., cerebral cortex, hippocampus, hypothalamus, thalamic nuclei, striatum, substantia nigra, and cerebellum, being involved in such processes as neurotransmission, plasticity, neuronal excitability, neuroinflammation and control of motor activity [12, 13, 14, 15].

TRPV1 is a nonselective cation channel, which is highly permeable to calcium. It was cloned in 1997 from rat dorsal root ganglia. TRPV1 ion channels are activated by the vanilloids capsaicin and resiniferatoxin [10]. The latter is an irritant diterpene found in the latex of several members of the genus *Euphorbia* such as *Euphorbia resinifera*, *E. unispina and E. poisonii* [16, 17]. Capsaicin and resiniferatoxin have in common a homovanillyl moiety essential for biological activity. These vanilloids are the most potent and specific agonists of TRPV1 ion chanels [20]. Studies have also indicated that the diterpene resiniferatoxin functions as an ultrapotent capsaicin analog being 200 fold more potent than capsaicin in depleting specific resiniferatoxin binding sites from rat spinal cord [18, 19].

TRPV1 nociceptors function as a polymodal noxious signal detectors that respond to a variety of noxious chemical and physical stimuli such as heat (≥ 43 °C, protons (low pH < 6.0), bradykinins, lipoxygenase products of arachidonic acid, and the endogenous lipid cannabinoid ligands *N*-arachidonoyl-ethanolamine (anandamide), and *N*-arachidonoyl-dopamine [10, 20, 21]. However, it is believed that each of these ligands because of their low potency and efficacy may not be able to stimulate the TRPV1 ion channel in vivo. Rather, it is likely that a number of the TRPV1 receptor ligands released during pathological states, will work in concert to induce opening of the ion channel [12]. Moreover, the functioning of the TRPV1 channel will be governed not only by the ligand but also by the TRPV1 milieu. TRPV1 activation thus reflects the integration of the impact of many different ligands and their timing [22].

In this review, we provided a concise summary of the actions of capsaicin on sensory receptors, the mechanisms that regulate the vanilloid receptor TRPV1 which is the molecular site of action for capsaicin. We also discussed the bioavailability of capsaicin after different routes of administration. We then concentrated on the in vivo and in vitro experimental research and clinical studies pertaining to the beneficial effects of capsaicin or TRPV1 in different disease states such as ischaemic/reperfusion brain injury, excitotoxic neuronal injury, Parkinson’s and Alzheimer’s diseases, systemic inflammation/sepsis and epilepsy. We illustrated those pathways by which capsaicin exerts its neuronal protective effects. Moreover, the results of studies that have suggested a neuroprotective potential for dihydrocapsaicin or a benefit of hot peppers in diet for human neuroprotection were discussed.

## Methods

We searched for peer-reviewed papers using a PubMed, Web of science, ScienceDirect, Scopus and Google Scholar search. We also searched the reference lists for relevant papers. Studies were evaluated based on their scientific merits.

### The Action of Capsaicin on Sensory Neurons

#### Sensory Nerve Stimulation by Capsaicin

The TRPV1-expressing sensory nerve terminals are excited when capsaicin is given in doses of µg/kg in vivo or applied locally in 0.03–10 µM concentrations in vitro [8, 23]. These fibers transmit nociceptive information arising from viscera and the skin to the central nervous system. In addition, activation of sensory nerves by capsaicin evokes the local release of the neuropeptides calcitonin gene-related peptide (CGRP), the tachykinins, substance P (SP) and neurokinin A (NKA) and somatostatin not only into the spinal cord but also in the periphery. These neuropeptides result in local tissue vascular responses, increased microvascular permeability, plasma extravasation and neurogenic inflammation. The afferent signals can also affect peripheral tissue functions by activating organ-specific reflexes or through the release of hormones. This has been termed the dual “sensory efferent” function mediated by the capsaicin-sensitive sensory neurons [5, 6, 7]. Moreover, a systemic “sensocrine” function for somatostatin released from sensory nerve terminals following the activation of peripheral nociceptors was proposed by Szolcsányi et al. [24]. Somatostatin which enters the circulation elicits systemic anti-inflammatory response by binding to G-protein coupled membrane receptors sst1–sst5 and decreasing the release of pro-inflammatory neuropeptides from sensory nerve endings as well as by acting on vascular endothelial, immune and inflammatory cells [24, 25]. TRPV1 stimulation by capsaicin has been demonstrated to protect the gastric mucosa from noxious injury. Studies have shown that in the rat stomach, stimulation of sensory nerve terminals by intragastric capsaicin (0.13 µM–160 µM) or resiniferatoxin (95.4 nM–0.29 µM) protected against experimental gastric mucosal damage evoked by ligating the pylorus (Shay ulcer), ethanol, acidified aspirin and other ulcerogenic agents. Gastroprotection by capsaicin-type agents involves an enhancement of the microcirculation and inhibition of gastric acid secretion mediated by the release of peptides from the sensory nerve terminals [26, 27, 28, 29, 30, 31].

Capsaicin has been used as a useful tool for studying pain pathways [8] Migraine is one of the most distressing neurovascular nociceptive pain in which the activation of meningeal afferents results in neuropeptide release e.g., CGRP and SP, mast cell degranulation, neurogenic inflammation and plasma leakage in dura mater. Neurogenic inflammation in turn causes the sensitization of the perivascular nociceptors and central nociceptive neurons in the trigeminovascular system and the development of neurovascular headaches [32]. Stimulation of TRPV1 channels located at trigeminal meningeal afferent nerve terminals has been shown to represent an in vitro migraine model for the study of trigeminal pain. It has been demonstrated that the application of 1, 2 and 10 µM capsaicin to nervus spinosus, a branch of the trigeminal nerve that innervates tissues around the medial meningeal artery caused increased nociceptive firing, which could be prevented by the TRPV1 receptor antagonist capsazepine [33]. Sensory nerve fibers containing CGRP immunoreactivity have been demonstrated in the cerebral vascular and in the trigeminal ganglion whereas capsaicin desensitization carried out in vivo and in vitro resulted in depletion of CGRP from cerebral arteries [34, 35]. Capsaicin-sensitive sensory nerves and CGRP have been implicated in pathophysiological mechanisms of migraine such as cerebral and dural blood vessel dilatation, stimulation of nociceptive trigeminovascular pathway, and mast cell degranulation [36, 37]. Activation of 5-HT3 receptors by serotonin leads to the release of CGRP and robust nociceptive firing from peripheral meningeal terminals of the trigeminal nerve [38], suggesting a role for this receptor in development of neurovascular headache. Studies have also shown that capsaicin stimulates CGRP release from trigeminal meningeal afferents, trigeminal ganglion preparations. Capsazepine prevented this effect of capsaicin [39], thereby, lending further support to the role played by TRPV1 in the initiation of migraine and other neurovascular headaches.

### Capsaicin-Induce Sensory Desensitization

The initial application of capsaicin will result in initial short-lasting stimulation of sensory receptors. After repeated or prolonged application of capsaicin [40] or resinaferatoxin [33], there will be reduced or even absence of neuronal response to subsequent nociceptive stimuli (functional desensitization) and to capsaicin itself (pharmacological desensitization). This capsaicin-induced “desensitization” is calcium-dependent and caused by prolonged gating of the TRPV1 cation channels [4]. The phenomenon of capsaicin-induced desensitization has been demonstrated in the eye, nasal mucosa, tongue, gastric mucosa, urinary bladder.

In the rat eye, desensitization was achieved after capsaicin concentrations of 0.03–0.15 mM as indicated by reduction of the eye-wiping response to irritation by homovanillyl octylester or zingerone. The effect lasted for 2 h. Capsaicin applied at 33 mM (1%) resulted in full desensitization that lasted for days [41]. On the other hand, when capsaicin (25 µg) was injected into the pre-optic area of the rat anterior hypothalamus a fall in rectal temperature occurred. This capsaicin-induced hypothermic response subsided after repeated application of capsaicin [42].

In the skin and mucous membranes, desensitization of TRPV1 capsaicin receptors on sensory nerve terminals was achieved by topical application of 1% capsaicin. This effect persisted for hours or days [8, 41]. In rat gastric mucosa, desensitization as indicated by loss of sensory neuron-mediated mucosal protection against noxious chemical challenge, was observed in rats treated with systemic capsaicin (150 or 180 mg/kg; 491.1 or 589.3 µmol/kg) [26] 2 weeks earlier or in animals given intragastric application of 1 or 2% (3.3 mM or 6.5 mM ) capsaicin [28, 30, 31]. Capsaicin or resiniferatoxin has been shown to exert vasodilator effects and increase gastric blood flow. Thus, resiniferatoxin (0.08–1.6 nmol/kg) injected into the rat jugular vein produced a marked and dose-dependent increase in gastric blood flow, while topical application of capsaicin (0.33–33 µM) or resiniferatoxin (0.16–1.6 µM) to the serosal surface of the stomach or jejunum resulted in a pronounced and long-lasting increase in blood flow. It has been demonstrated that this increase in gastric blood flow and changes in blood pressure elicited by i.v. or local application of capsaicin or resiniferatoxin were absent in rats desensitized earlier with systemic capsaicin treatment. The desensitization was achieved by s.c. administration of 30 + 60 + 90 mg/kg of capsaicin, given on 3 consecutive days; the last dose was given 1–2 days before the experiment [43].

Capsaicin desensitization has also been demonstrated in rat urinary bladder in vitro, with low concentrations of capsaicin which stimulate primary afferent terminals. When capsaicin (0.1–1.0 µmol/1) was applied for 5 min, the release of immunoreactive CGRP induced by a second exposure to capsaicin was reduced [44]. In anesthetized rats, desensitization of urinary bladder (increased volume threshold to micturition and decreased contractile response to 1.0–10 µM capsaicin) was reported after serosal application of capsaicin (0.03-10.0 µM) or resiniferatoxin (0.1 or 10.0 nM) for 15–30 min [18]. Desensitization of rat bladder sensory afferents was also achieved by intravesical instillation of capsaicin (10–100 µmol) or resiniferatioxin (10–100 nmol) with the lower concentrations of each vanilloid resulting in partial desensitization as opposed to complete and long lasting desensitization by the higher concentrations. Resiniferatioxin was found approximately 1000 times more potent than capsaicin in achieving bladder sensory desensitization [45]. In humans, desensitization of bladder sensory afferents was employed to treat detrusor hyperreflexia using intravesical resiniferatoxin (50 or 100 nM in 100 ml solution) [46].

Human nasal mucosa was desensitized by repeated application of 50 µl capsaicin (50 nmol) to one nostril once a day for 5–7 days [47]. In humans who are chilli likers or users, Rozin et al. [48] reported the presence of a very small, yet a reliable and significant decline in the sensitivity to capsaicin, suggesting that a slight desensitization effect have developed. Capsaicin may induce sensory desensitization by altering cellular excitability by blocking voltage-gated Na^+^ or voltage-sensitive Ca^++^ channels [49, 50], depleting sensory nerve terminals of their content of neuropeptides, or blocking axonal transport [49, 51]. Sensory desensitization depends not on excessive stimulation of the receptor but rather on the TRPV1 agonist’s robust binding and prolonged opening of the ion channel [20, 30].

The phenomenon of capsaicin desensitization was the basis for the use of topical capsaicin formulations in the treatment of painful neuropathic conditions e.g., post-herpetic neuralgia, diabetic neuropathy, and polyneuropathy associated with human immunodeficiency virus [52, 53], nonallergic (vasomotor) rhinitis [54], as well as in dermatological conditions such as pruritus ani, and pruritus of hemodialysis [55]. Moreover, capsaicin or resiniferatoxin were used to treat a number of troublesome urological disorders e.g., neurogenic bladder, overactive bladder, and painful bladder syndrome [56].

### The Neurotoxic Effects of Capsaicin

With high doses/concentrations of capsaicin, sensory neuron blocking and neurotoxic effects on polymodal nociceptors and other capsaicin-sensitive chemoceptors were described after perineual, intrathecal, topical, or systemic treatment in the adult and neonate rats [8, 9, 20]. The sensory neuron blocking action of capsaicin is the term used to describe a state of functional desensitization of afferent sensory receptors that can last for days. There is no release of sensory mediators. On the other hand, long-term neurotoxic effects are produced by local application of high concentrations (e.g., perineural 1% solution) or treatment of rats with increasing doses of capsaicin (from 30 to 50 mg/kg s.c. up to a total dose of 120–300 mg/kg) over the course 2–4 days [8, 57]. In these animals, there is degeneration of peripheral and central terminals and axons or even irreversible cell death of capsaicin sensitive primary afferent neurons besides depletion of their SP and CGRP peptide content from peripheral terminals and sensory neurons [20, 41].

### The Bioavailability of Capsaicin

#### Gastrointestinal Absorption of Capsaicin

Studies have demonstrated that capsaicinoids are rapidly absorbed from the stomach and small intestine. According to Monsereenusorn’s [58] research, the amount of capsaicin absorbed in vitro from rat and hamster intestinal sacs was proportional to its concentrations in the mucosal medium. In the rat, Kawada et al. [59] have reported gastrointestinal absorption of 50% of an orally administered capsaicin dose of 3 mg (∼13.5 mg/kg) suspended in basal diet within 1 h. Only 15% of capsaicin remained in gut by 3 h after dosing. The study suggested that capsaicin and/or dihydrocapsaicin were partly subjected to metabolism during absorption in jejunum, transported to the portal vein and then metabolized in the liver.

In their study in anesthetized rats, Donnerer et al. [60] reported rapid rate of capsaicin absorption from the jejunum and ileum than from the stomach. The authors found in pylorus-ligated rats that 15 min after intragastric application of capsaicin or DHC (50–500 µg/ml), 30–50% was absorbed, with almost no degradation occurring in the stomach. In contrast, in rats with open pylorus, ∼ 90% of 50 µg/ml and 75% of 500 µg/ml capsaicin were absorbed from the intestine, with considerable degradation taking place in the intestine. Moreover, [3H]-dihydrocapsaicin ([3H]-DHC) and unlabelled capsaicin were almost completely metabolized before reaching the general circulation. Capsaicin was detected in the portal vein blood in the range of 50 and 200 ng/ml (75 nM–0.3 µM) after 500 µg/ml intragastric capsaicin.

Other researchers reported that after an orally administered dose of 30 mg capsaicin in rats, 1.24% was detected in blood, liver, kidney and intestine in 1 h and 24.4% was detected in these tissues at 24 h, with less than 0.1% being excreted in urine. Capsaicin concentrations in serum and liver reached 90 µg/ml and 45 µg/ml at 1 and 3 h, respectively [61]. In this context, it is worthy to mention that the rat hepatobiliary tract receives dense innervations of CGRP-containing nerve fibers, suggesting the involvement of these peptidergic visceral afferents in the regulation of hepatobiliary activities and vascular haemodynamics [62]. Moreover, capsaicin given orally at 10–1000 µg/kg conferred protection against the hepatotoxic effects of carbon tetrachloride in rats, ameliorating the rise in serum aminotransferases, liver histologic necrosis and preventing the decrease in glycogen synthesis as well as in DNA contents in hepatic nuclei caused by the toxicant [63]. This suggests that orally given capsaicin can reach the liver and exerts hepatic protective effects, which may involve the release of vasodilator peptides from sensory nerve endings.

Capsaicin has also been shown to be readily excreted into bile following systemic or gastrointestinal administration. Thus, after intragastric or intraduodenal application of capsaicin (4–400 µg/ml; 10–1000 µg/kg), capsaicin was readily excreted into bile reaching peak levels of 100–248 and 144–698.6 ng/ml 75 or 60 min, respectively. Capsaicin levels in bile after its intraduodenal application were high after 60 min and then decreased, with an elimination half-life of approximately 22.5–26.3 min which suggests rapid elimination of capsaicin from bile. Meanwhile, capsaicin was detected in bile at concentration of 86 and 75 ng/ml 15 min after i.v. administration of 10 µg/kg. These findings suggest that negligible amount of capsaicin is secreted into bile from the systemic circulation while the majority of capsaicin reaches bile *via* the portal vein from the gastrointestinal tract [64]. Nevertheless, it is clear that capsaicin can reach bile at concentrations that have excitatory effects on sensory nerve endings and modulate bile secretion.

### Systemic Administration of Capsaicin

Capsaicin has been shown to be readily distributed to tissues following its systemic administration. According to Donnerer et al. [60], approximately 50% intact capsaicin was found in trunk blood and the brain of rats three minutes after receiving i.v. capsaicin at a dose of 2 mg/kg or 90 min following s.c. injection of 50 mg/kg capsaicin. In contrast, less than 5% of intact drug was found in trunk blood and in brain 15 min following intragastric application. Moreover, studies by Saria et al. [65, 66] have demonstrated a rapid entrance of capsaicin into the central nervous system after i.v. administration of 2 mg/kg of the drug in rats. Capsaicin was detected at ng/g concentrations in brain, liver and blood with a 5-fold higher concentration in brain and spinal cord and 3-fold higher value in the liver than in blood. Three minutes after i.v. injection, capsaicin concentrations in brain, liver and blood were 2763, 1736 and 581 ng/g, respectively. Capsaicin levels fell rapidly in liver and blood and liver 10 min later, as it was reallocated to adipose tissue and/or became inactivated due to formation of conjugates. The above observations suggest that capsaicin given *via* systemic routes can reach the brain and other tissues at concentrations in the range of ng/g. Capsaicin at these concentrations has been demonstrated to exert a powerful excitatory effect on capsaicin-sensitive peripheral sensory nerve endings and can excite brain TRPV1 receptors [8, 20]. Indeed, stimulation of these channel receptors in brain has been shown to modulate of neuronal activity [67], release of synaptic neurotransmitters [68, 69] and/or neuropeptides e.g., substance P, somatostatin and CGRP [36].

### Capsaicin in Small Doses is Neuroprotective

#### Brain Ischaemic/Reperfusion Injury

Capsaicin has been shown to prevent neuronal death and neurological disability that followed experimental brain ischaemic/reperfusion (I/R) injury *via* inducing hypothermic response [70], preventing oxidative stress, release of proinflammatory mediators and inhibiting neuronal apoptosis [72, 74] or by direct action of blood vessels resulting in improving myotonic tone and causing vasoldilataion [78, 79]. Thus, Pegorini et al. [70] have demonstrated increased survival of pyramidal neurons in the hippocampus in repose to systemically administered capsaicin (0.05–0.6 mg/kg s.c.) in a gerbil model of global cerebral I/R damage. Capsaicin was administered five minutes after reperfusion. The number of surviving neurons increased by 80% at 7 days after ischaemia in the group given 0.2 mg/kg of capsaicin, which also stopped the ischemic group’s rise in motor activity and cognitive deterioration. The capsaicin’s effects are likely to involve TRPV1 stimulation being lessened by co-administering the specific TRPV1 antagonist capsazepine (0.01 mg/kg). One suggested mechanism underlying neuroprotection by capsaicin was a decrease in body temperature. The latter has been demonstrated to be neuroprotective in experimental models of brain ischaemia [71].

Capsaicin’s neuroprotective action was also evident when given 30 min before initiating brain ischaemia. In global cerebral transient I/R rat model, intraperitoneal (i.p.) capsaicin (1 or 2 mg/kg) suppressed the I/R-induced rise in brain lipid peroxidation and nitric oxide. It also prevented the decline in paraoxonase-1 activity and interleukin-10. A concomitant reduction of the increase in glial fibrillary acidic protein (GFAP) following I/R was also evident. On histology, a decrease in neuronal degeneration in cerebral cortex and substantia nigra as well an increase in hippocampal granular cell layer thickness was observed after capsaicin treatment. Capsaicin thus prevents neuronal injury by reducing oxidative stress and proinflammatory mediators possibly by inactivating astrocytes [72].

Moreover, the central administration of capsaicin was shown to prevent cerebral infarction in rats with middle cerebral artery blockage. Capsaicin 1 or 3 mol (1 µl) injected into the peri-infarct region, 30 min after reperfusion, resulted in 39% and 21% decrease in the infarct area, respectively. Behavioral testing showed a decrease in neurological impairments and delayed falling from rotarod [73].

Capsaicin may protect neurons *via* an antiapoptotic mechanism. In vitro, apoptotic cell death was induced in primary rat hippocampal neurons by hypoxia and subsequent re-oxygenation. Capsaicin (3–30 µmol/l) was demonstrated to prevent the production of more intracellular reactive oxygen species (ROS) and the activation of caspase-3, which may have been mediated by the PI3K/Act signaling pathway [74].

**Fig. 1:**
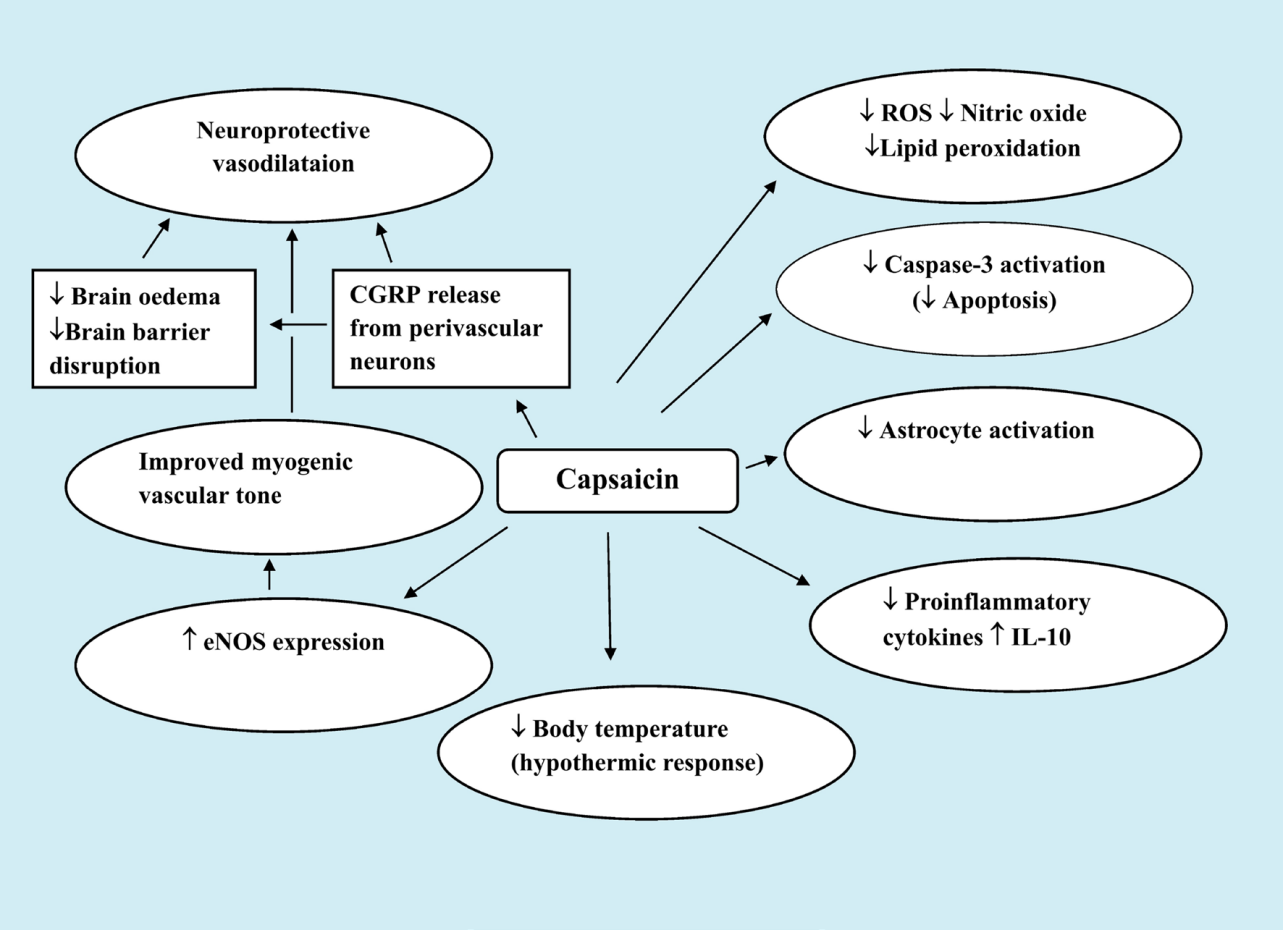
Mechanisms underlying protection by systemic administration of capsaicin in small doses in ischaemic/reperfusion brain injury

Capsaicin *via* the release of vasodilator peptides from sensory nerve terminals upon their stimulation exerts vasodilator effects [5, 6, 7, 43]. It has been demonstrated in vitro that capsaicin, CGRP, or substance P relaxed cerebral arteries that had previously constricted by neuropeptide Y. CGRP was the most effective in this respect [75]. This peptide has been shown to a potent vasodilator in a number of vascular beds [76]. It has also been demonstrated that CGRP could prevent brain oedema and blood-brain barrier breakdown during cerebral I/R [77]. Several studies have suggested that a vascular mechanism (s) may by underlying the effect of capsaicin in neuroprotection [78, 79]. In this context, Khatibi et al. [78] have shown that in 10-day-old rat pups with ischemia or hypoxic brain injury, prior administration of capsaicin (0.2 or 2 mg/kg, i.p.) reduced the infarct volume. Capsaicin was also demonstrated to improve the myogenic tone in middle cerebral artery segments. The authors suggested that the improvement in vascular dynamics have a role in reducing the severity of brain tissue injury by capsaicin. The work by Xu et al. [79] has also demonstrated a vascular pathway for capsaicin-induced neuroprotection. In their research, it was found that capsaicin helped spontaneously hypertensive rats live longer and postponed the onset of cerebral stroke. Until a stroke occurred, these animals were given chow with 0.02% capsaicin. Capsaicin in the diet increased TRPV1 expression and eNOS levels in carotid arteries. Capsaicin enhanced the endothelium-dependent relaxation of basilar arteries. This action was antagonized by capsazepine. Additionally, capsaicin reduced the intima-media thickness of the intracranial arteries. These vascular effects of capsaicin described in the aforementioned studies could represent an important mechanism by which capsaicin/TRPV1 contribute to the prevention of vascular disease (Fig. 1).

**Fig. 2:**
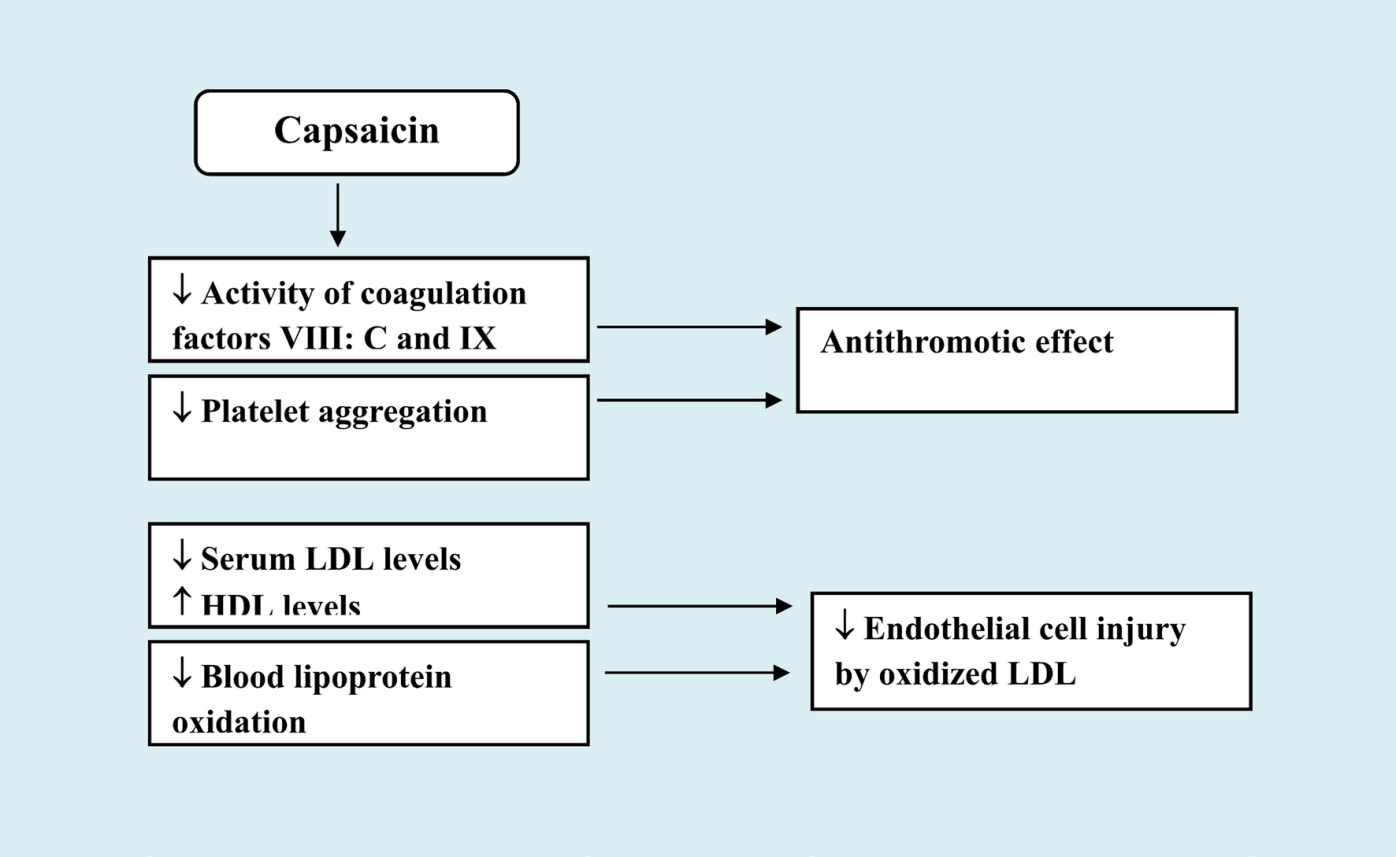
Favourable effects of capsaicin administration in cerebrovascular disease

The aforementioned studies suggest therefore that capsaicin’s vasodilator effects may help shield brain tissue from ischemic damage. Capsaicin or peptides released from nearby perivascular neurons in response to capsaicin stimulation may offer neuroprotective vasodilatation. Moreover, there is evidence from an in vitro study that capsaicin or dihydrocapsaicin (DHC) was able to prevent platelet aggregation and blood coagulation. In this study by Adams et al. [80], capsaicin (25–100 µmol/l) or dihydrocapsaicin (DHC) (6.25–100 µmol/l) inhibited platelet aggregation in venous whole blood and the activity of coagulation factors VIII: C and IX in plasma from healthy subjects. These observations represented another and novel mechanism by which capsaicin or (TRPV1 stimulation) *via* antithrombotic action protects against cerebral or coronary vascular disease (Fig. 2).

### Capsaicin’s Favorable Effects on Lipid Profile

Hyperlipidaemia is a well established important risk factor for cerebral stroke and drug treatment of hyperlipidaemic disorders with lipid-lowering agents aims to decrease low-density lipoprotein (LDL-C) and also increase high-density lipoprotein (HDL-C) levels [81]. In this context, there is evidence to suggest that capsaicin may also act to reduce the likelihood of stroke *via* effects on lipid metabolism. Lee et al. [82] have shown that rats given capsaicin orally (3 mg/kg every day for 3 days) had lower serum LDL levels, but higher levels of HDL and triglycerides. Additionally, there was a decline in lipid peroxidation in the liver, lung, kidney, and muscle. Moreover, an i*n vitro* study by Chen et al. [83]. have shown that oxidized low-density lipoprotein-induced injury of human umbilical vein endothelial cells was prevented by capsaicin as indicated by a reduction in cytochrome c production, caspase-3 activation, and chromosomal condensation. It has also been shown that capsaicin administration in I/R cerebral damage prevented the decline in brain PON-1 activity [72]. This enzyme is endued with antioxidative action and in circulation acts to prevent oxidation of LDL [84]. The above studies therefore suggest that capsaicin by decreasing serum LDL levels as well as *via* antiinflammatory and antioxidative actions will have beneficial effects by interfering with the progression of atherosclerotic vascular disease.

Other researchers suggested that *via* preventing the oxidation of HDL in the blood, capsaicin (from chilli peppers) in the diet may lessen the risk of stroke. Participants in the Ahuja and Ball trial [85] consumed a plain or chilli-based diet for 4 weeks. Thus, 30 g of freshly chopped chilli pepper mixture (including 55% cayenne pepper) were consumed each day. Total cholesterol, HDL, LDL, triglycerides, and overall antioxidant status were found to be unaffected by the two dietary approaches. However, regular use of chilli was linked to a 10.4% decreased rate of blood lipoprotein oxidation (in comparison to the bland diet). It should be noted, however, that, the *Capsicum* fruit has rich content of antioxidant compounds like beta-carotene, ascorbic acid (vitamin C) and alpha-tocopherol (vitamin E) [86, 87] which could beside capsaicin have played a significant role in these effects of the consumed chilli pepper mixture (Fig. 2).

### The Neuroprotective Effects of Dihydrocapsaicin

Studies have also revealed that dihydrocapsaicin (DHC) has neuroprotective qualities. This pungent capsaicinoid is present in hot peppers in significant amounts. In the rat, DHC (0.5–10 mg/kg s.c.), like capsaicin, caused hypothermia in rats and demonstrated desensitization and cross-tolerance to the hypothermic effects of capsaicin. Additionally, it caused substance P to be depleted from the dorsal spinal cord and dorsal root ganglia [88]. In their study, Cao et al. [89] have demonstrated a neuroprotective effect for DHC (1.25 mg/kg/h twice over two days) against cerebral I/R injury in mice. The effect was TRPV1 receptor-dependent and involved reducing body temperature. This is because mice kept under normothermic conditions or TRPV1 knockout animals (normothermic) were not protected by DHC from I/R damage. In a different investigation by Janyou et al. [90], DHC (5 mg/kg and 10 mg/kg, i.p.) given 15 min before reperfusion protected against cerebral I/R injury in the rat. The study found a significant reduction in neurological impairments and infarct volume. With regard to the mechanisms involved, DHC has been shown to inhibit oxidative stress, release of inflammatory mediators and neuronal apoptosis as well as reduced blood-brain barrier disruption. However, DHC showed no effect on cerebral blood flow or body core temperature in this study. The above studies therefore suggest that DHC like capsaicin has a neuroprotective potential. Yet, there is a clear need for further studies to delineate the exact mechanism involved.

### Excitotoxic Neuronal Injury

Capsaicin has been shown to protect cultured AF5 rat mesencephalic cells from *N*-methyl-D-aspartate (NMDA) toxicity in vitro. Capsaicin application (1–10 µM) for 20 min prior to NMDA led to an increase in cell vitality (MTT assay). However, the protective action of capsaicin appears not to be mediated *via* TRPV1 mechanisms since it was not inhibited by the selective TRPV1 antagonist capsazepine (10 µM) [91]. In a different experiment, it was found that pretreatment with 3 or 10 µmol of capsaicin protected rat cortical neurons from glutamate toxicity, decreasing calcium influx, and the release of lactic dehydrogenase (LDH) into the medium. Capsaicin, on the other hand, failed to prevent glutamate-induced cell death in cultured cortical neurons from TRPV1 mutant mice. This indicated that protection requires TRPV1. The study also hypothesized that down-regulation of NMDA receptors may play a role in capsaicin’s ability to protect neurons. Moreover, in he study by Sakamoto et al. [92] TRPV1 activation by capsaicin (50 nmol/eye) has been shown to almost totally prevent ganglion cell death in rats receiving intra-vitreal injections of NMDA (in retina). Capsaicin’s effects were preventable by capsazepine, CGRP receptor antagonist or tachykinin NK1 receptor antagonist. These observations suggest that the release of CGRP and takykinins by TRPV1 was the mechanism responsible for this protective action of capsaicin.

More recently, Klinic et al. [93] have reported a protective effect for capsaicin (0.2-5-mg/kg, i.p.) against ibotenate-induced excitotoxic neonatal brain injury in rat pubs, improving cerebral white matter and gray matter alterations. These effects involved inhibition of mast cell activation and the release of pro-inflammatory cytokines IL-1β, and IL-6 and neuronal survival factor activin A in brain. Previous work revealed that mast cells are activated in immature brain if rats early after ischaemic/hypoxic injury [94] and these cells have a role in the neuronal damage in I/R neuronal damage *via* inflammatory mediators release [95].

The above studies, therefore, suggest that capsaicin protects against excitotoxic neuronal injury via mechanisms that is likely to involve TRPV1 activation and inhibition of inflammatory mediator release from mast cells.

### Parkinson’s Disease

Parkinson’s disease is caused by a progressive death of the pigmented dopaminergic neurons in the substantia nigra compacta (SNc) of the midbrain basal ganglia [96]. The basal ganglia adjust actions started in the cerebral cortex by cortico–basal ganglia–thalamo–cortical loops [97]. Critical levels of dopamine depletion in the SNc and striatum cause the cardinal features of bradykinesia, stiffness, postural instability, and hand tremors [98]. In Parkinson’s disease, 95% of cases are sporadic [99] and it is generally agreed that environmental contaminants, including pesticides, in addition to hereditary predisposition are responsible for the death of dopaminergic cells [100, 101]. Oxidative stress and inflammation are the main causes of dopaminergic cell death [102, 103]. Parkinson’s disease can be induced in experimental animals with nigrostriatal toxins such as 1-methyl-4-phenyl-1,2,5,6-tetrahydropyridine (MPTP), its metabolite 1-methyl-4-pyridinium (MPP+) or rotenone, a naturally occurring pesticide and a mitochondrial complex I (NADH-quinone oxidoreductase) inhibitor [104].

Capsaicin has been shown to lessen the reduction in tyrosine hydroxylase (TH)-positive cell bodies in SNc, striatal fibres and striatal dopamine levels as a result of MPTP injection in rats. Capsaicin (0.5 mg/kg, i.v.) increased the latency to fall from a rotarod while improving motor balance. This neuroprotective action involved TRPV1 activation being counteracted by pretreatment with the specific TRPV1 antagonists capsazepine and iodoresiniferatoxin (1 mg/kg, i.p.) [105]. According to the study findings, capsaicin exerts its neuroprotective benefits by blocking astroglial activation and the subsequent release of reactive oxygen species, tumour necrosis factor alpha (TNF-alpha), and interleukin-1beta (IL-1beta), which are inflammatory cytokines.

Other studies by Nam et al. [106] demonstrated protection by capsaicin against MPP + or adeno-associated virus α-synuclein-induced Parkinson’s disease in rats. Capsaicin (1 mg/kg, i.p.) given on daily basis for 7 days prior to MPP + or for 7 weeks before α-synuclein prevented the neurodegeneration. An increase in TH + neurons in SNc and TH + fibres in the striatum was also detected. Inactivation of TRPV1 using lentivirus with interference DNA directed against TRPV1 was found to reduce the capsaicin effect. This implied a role for the TRPV1 channel in the neuroprotection by the vanilloid. The study suggested that TRPV1 activation by capsaicin causes the release of ciliary neurotrophic factor from astrocytes that express TRPV1 receptors which afforded the neuroprotection.

Another work by Park et al. [107] revealed that capsaicin may prevent MPP+-induced dopaminergic neuron death by preventing the generation of reactive oxygen metabolites caused by microglial activation *via* TRPV1.

These aforementioned results showed that in animal models of Parkinson’s disease, capsaicin given *via* systemic route was able to effectively prevent dopaminergic cell loss. It is likely that antioxidant action besides anti-inflammatory effect via inhibiting astroglia cell-mediated release of inflammatory mediators have contributed to the observed protection.

**Fig. 3:**
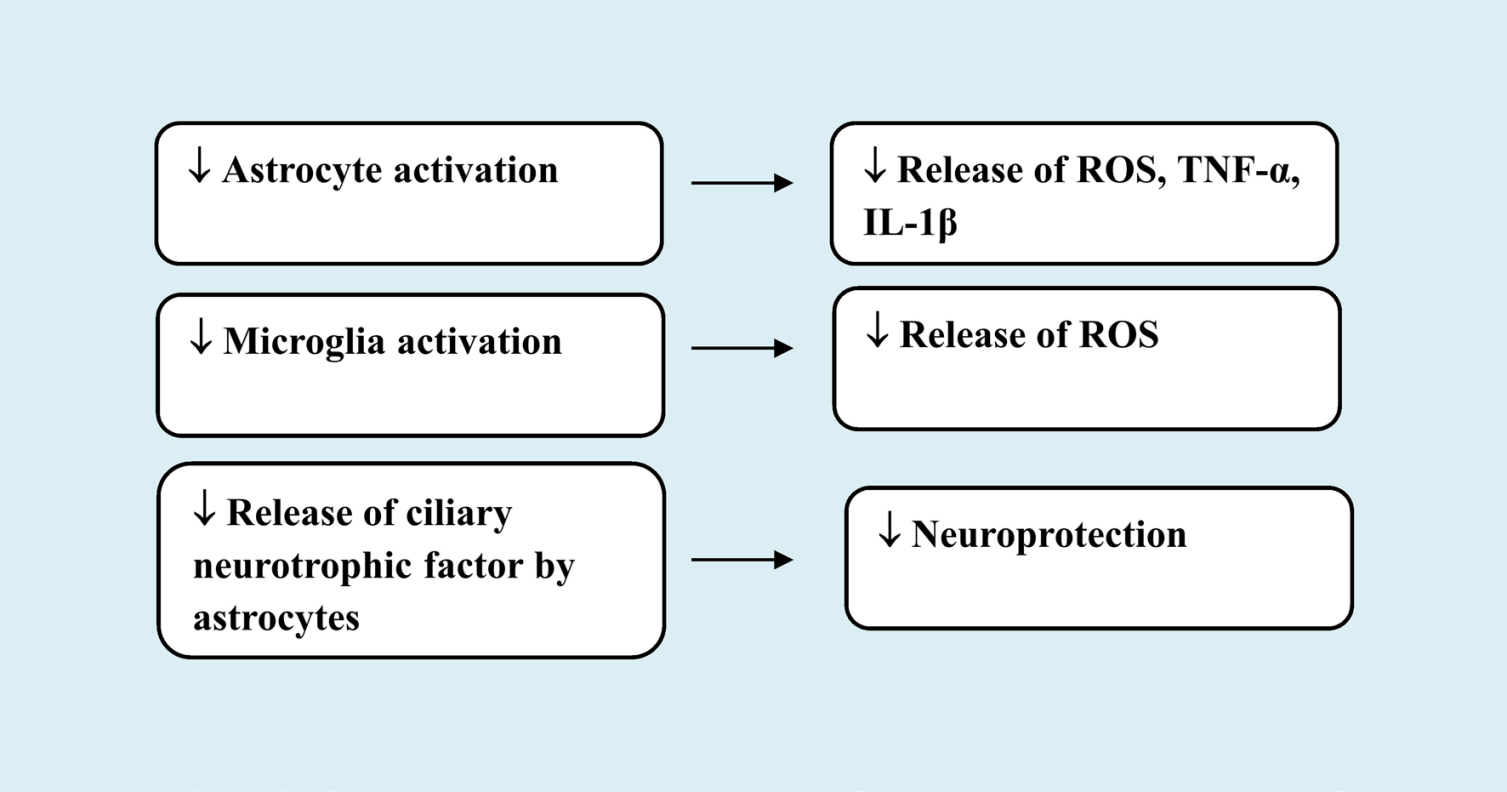
Protective mechanisms of small dose capsaicin in experimental Parkinson’s disease

Interest in botanical medicine is on the rise and in looking for new remedies that may interfere with neurodegeneration, botanicals may prove valuable candidates [108, 109]. In this context, it has been shown that a methanolic extract of hot pepper (capsaicin content 1.2%). was capable of reducing the extent of damage to cortical neurons and astrocytes during severe hypoglycaemia in mice brain by interfering with oxidative stress and 5-lipoxygenase activation [110]. Furthermore, the extract of hot peppers afforded protection against the degeneration of dopaminergic cells evoked by rotenone injections in mice. The increase in brain lipid peroxidation, nitric oxide, and depletion of reduced glutathione produced by rotenone were alleviated by the extract. It also reduced 5-lipoxygenase and the decrease in butyrylcholinesterase activity in brain of rotenone treated animals and provided protection from the histopathological alterations caused by rotenone, namely the decrease in number and degeneration of SNc neurons, neuronal degeneration in cerebral cortex and hippocampus and the decrease in glial fibrillary acidic protein (GFAP)-positive astrocytes [111]. These results suggest that hot peppers in diet may prove of benefit in combating neurodegeneration by virtue of anti-oxidative and anti-inflammatory actions (Fig. 3).

### Alzheimer’s Disease

This devastating disease which impairs memory and cognitive function is the leading cause in elderly dementia. The disease starts off with an insidious onset of loss of memory for recent events/episodic memory, before progressing over time to the point where one is unable to do even the most basic daily activities [112, 113]. The presence of extracellular amyloid deposits or senile plaques, neurofibrillary tangles made of hyperphosphorylated microtubule associated protein (tau) within neurons, synaptic loss, neuronal death as well as regional brain atrophy that involves cerebral cortex, entorhinal cortex and hipocampal formation constitute the main neuropathological features of Alzheimer’s disease. The existence of amyloid-β peptide (Aβ_40−42_) deposits—a byproduct of amyloid precursor protein (APP) cleavage by the proteases β- and γ secretases, is considered a critical event in the disease process, initiating oxidative stress, neuroinflammtion and neurodegeneration [114, 115].

There is evidence that dietary and lifestyle choices can affect this disease’s risk, most likely through affecting such processes as free radical mediated oxidative damage and neuroinflammation [116, 117]. A number of studies have investigated the effect of capsaicin/*capsicum* on cognitive functioning. In humans, two studies based on telephone surveys have provided contrasting results. In the study by Liu et al. [118], it was found that capsaicin in diet (chilli pepper) had an inverse relationship with Aβ_40_ and total Aβ levels in serum (but not with Aβ_42_). The study’s findings also suggested that people who were 40 years of age or older would benefit from consuming *Capsicum* since it would improve their cognitive abilities. Shi et al. [119] study, revealed, however, that increasing chilli intake may accelerate cognitive impairment. The performance of the participants’ memory was rated as good, very good, OK, bad, extremely bad. According to the study’s findings, participants who consume more than 50 g of chilli per day had self-reported increasingly poor memory and memory deterioration compared to non-consumers. However, it would be useful to examine the effect of known amount of capsaicin introduced into human diet on cognitive performance or at least to determine the capsaicin content in the chilli pepper for delineating a dose-response effect on memory.

Other studies in animals suggested that extracts of red peppers prevented the impairment in memory and reduces Aβ load in experimentally-induced Alzheimer’s disease. In the studies by Yang et al. [120], the authors looked at the effects of ethanolic red pepper extracts with various levels of pungency in diabetic rats given intrahippocampal infusions of Aβ_25–35_. Red pepper extracts were added to the diet and were the equivalent of a human consuming 3 g of pepper per day. High-pungency pepper extracts were observed to reduce tau phosphorylation, Aβ buildup, and memory impairment. The effect of hot pepper extract (25 and 50 mg/kg) was also examined in the model of Alzheimer’s disease caused by AlCl_3_ in the rat. AlCl_3_ was given i.p. to rats every day for two months. Beginning with the second month of AlCl_3_, rats were also given i.p. treatments with saline or *Capsicum* extract. Hot pepper extract reduced IL-6 and Aβ concentrations in the brain while decreasing AlCl_3_-induced oxidative brain damage. Hot pepper reduced memory loss, strengthened neuromuscular function, and protected neurons from damage by AlCl_3_. These results suggest that spicy peppers, a popular dietary ingredient, may be useful for preserving cognitive function and halting neurodegeneration in the Alzheimer’s disease brain [121].

It is worthy to mention here that the beneficial effect of chilli peppers in diet on memory described above, cannot be attributed solely to capsaicin. Ascorbic acid (vitamin C), alpha-tocopherol (vitamin E), phenolic compounds, anthocyanin, β-carotene, and other carotenoid pigments such as lutein, zeaxanthin, capsanthin, and capsorubin are among the other phytochemicals that chilli peppers are rich in besides their content of pungent capsaicin [1, 86, 87]. Some varieties of carotene-rich peppers contain 136 µg of β-carotene per gram fresh weight [86]. The phytochemicals in peppers have powerful antioxidant activities and may act in concert with capsaicin and/or other capsaicinoids, which makes these fruits attractive nutraceuticals.

The effect on Aβ load of supplementing diet with capsaicin was investigated in experimentally-induced Alzheimer’s disease in mice. Using APP/PS1 genetic mouse model of Alzheimer’s disease, Wang et al. [122] found that feeding mice chow supplemented with 0.01% capsaicin lowered the brain Aβ load by 32%. Additionally, mice receiving diets supplemented with capsaicin (estimated daily intake: 30 mg/kg) experienced a reduction in tau hyperphosphorylation, activated glia cells, levels of proinflammatory cytokines (TNF-α, IL-6, interferon-γ), neuronal apoptosis, learning disability, and impaired spatial memory. In vitro tests utilizing human neuroblastoma cells that over-express APP659 revealed a reduction in Aβ_42_ and Aβ_40_ levels in cell lysate with the addition of capsaicin (0.1–50 µM). This effect was dose-dependent. According to the study’s findings, it may be suggested that capsaicin prevents the synthesis of Aβ by directing APP processing towards cleavage by α-secretase.

Other researchers employed an Alzheimer’s disease model in which adult mice were injected with Aβ_42_ (100 µM; 2.5 µl/mouse) intracerebroventricularly (i.c.v.). Treatment with capsaicin (1 mg/kg/day, i.p.) decreased the animal’s impaired spatial learning and memory, synapse loss, and the impaired long-term potentiation in hippocampal CA1 area. Capsazepine (1 mg/kg, i.p.), however, had no effect on these measures in Aβ_42_-treated mice [123]. Therefore, it is not clear whether these effects are related to TRPV1 activation.

In patients with type 2 diabetes, Aβ aggregates, hyperphosphorylated tau, and ubiquitin were identified in beta cell islets [124]. In a study by Xu et al. [125], the researchers utilized rats fed a high-fat diet and injected i.p. with streptozotocin to simulate type-2 diabetes mellitus. For 12 weeks, rats fed a meal containing 0.01% capsaicin showed considerably lower levels of blood sugar, plasma insulin, and insulin resistance. Additionally, capsaicin decreased the hyperphosphorylation of the tau protein in hippocampus. These findings imply that use of capsaicin may reduce type-2 diabetic individuals’ risk of developing Alzheimer’s disease. Another study by Jiang et al. [126] demonstrated that intragastric administration of capsaicin (10 mg/kg) to rats 1 h prior to exposure to cold water stress was able to improve the animals’ dendritic regression, tau hyperphosphorylation, and loss of the memory-associated proteins PSD93 and synapsin I.

Moreover, data from the study by Balleza–Tapia et al. [127] showed that capsaicin was able to protect against the Aβ-induced impairment of gamma oscillations in vitro. The latter are crucial for memory functions and are diminished in Alzheimer’s disease [128, 129]. Capsaicin (10 µM) added to hippocampus slices incubated with Aβ_1–42_ significantly reduced kainic acid-evoked gamma oscillations. This effect of capsaicin was shown to be prevented by capsazepine (10 µM) and to be missing in TRPV1 knockout mice, indicating its mediation by stimulating TRPV1 channel receptors [127].

Capsaicin, however, may support the amyloidogenic pathway, according to certain other investigations. In contrast to control animals, Pákáski et al. [130] found a 1.7-fold increase in membrane-bound APP in the cerebral cortex of capsaicin-treated rats. The hypothesis put forth at the time was that capsaicin modifies APP metabolism, favouring the amyloidogenic pathway. In this experiment, rats were given sc. injections of 20 mg/kg capsaicin, followed by 30 mg/kg two days later. These doses are obviously very high and could have a desensitizing effect on TRPV1 receptors. Using human neuroblastoma SH-SY5Y cell line, Grimm et al. [131] found that applying capsaicin and DHC at 10 µM for 24 h raised Aβ-levels through processes including elevated β- and γ-secretase activity and impeded Aβ-degradation. Given that cells were exposed to capsaicin over an extended period of time, desensitization rather than activation of TRPV1 channel receptors may have caused the effects that were seen.

**Fig. 4:**
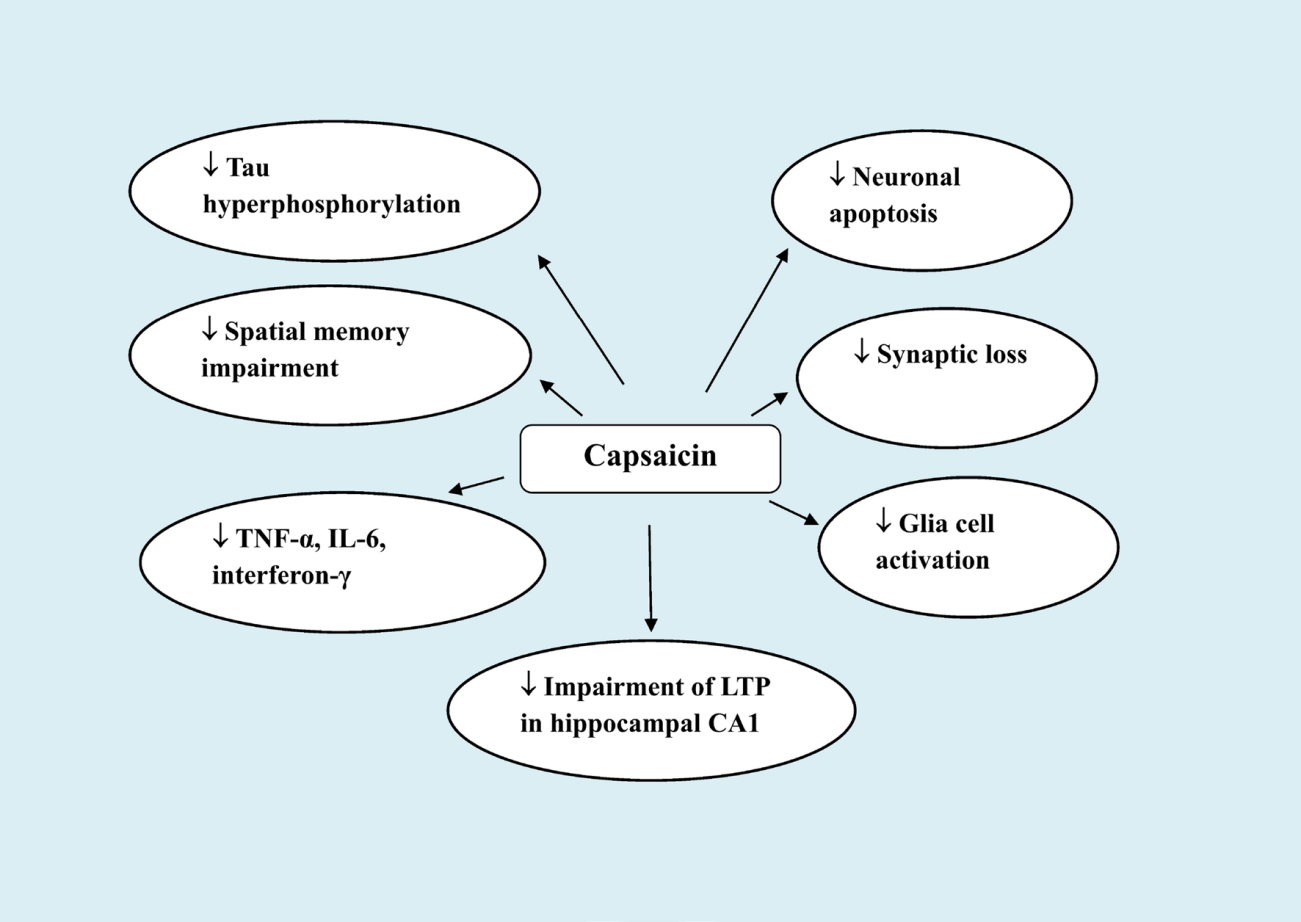
Effects of supplementing diet with capsaicin or systemic injection of small dose capsaicin in experimental Alzheimer’s disease

It is therefore clear that the effects of capsaicin/TRPV1 in terms of their ability to modulate tau hyperphosphorylation, Aβ aggregation and memory impairment in experimental Alzheimer’s disease are intriguing and suggest that TRPV1 may represent a novel pharmacological target in patients with Alzheimer’s disease. It is also likely that the effects of capsaicin or TRPV1 stimulation on inflammatory cells participate in part to inhibiting amyloidogenesis by decreasing the contributing role of an inflammatory and oxidative milieu (Fig. 4).

### Systemic Inflammation/Sepsis

Capsaicin has been shown to have positive antioxidant and anti-inflammatory properties when administered systemically during sepsis or mild systemic inflammation [132, 133]. In the studies by Demirbilek et al. [132] sepsis was induced in rats by caecal ligation and puncture. Capsaicin given s.c. at 1 mg/kg was found to lower TNF-α, IL-6, and nitric oxide while increasing IL-10 in plasma. Additionally, rats with sepsis given capsaicin had higher levels of superoxide dismutase and decreased lipid peroxidation in their liver and lung compared to controls. These effects were not observed in septic rats with systemic capsaicin-induced sensory deafferentation (capsaicin 150 mg/kg injected sc. over 3 consecutive days). These observations therefore provides evidence for a protective role of capsaicin-sensitive sensory nerves during sepsis.

In rodents, injection of lipopolysaccharide (LPS), a component of the outer wall of Gram negative bacteria, induces oxidative stress and the release of pro-inflammatory cytokines such as tumour necrosis factor-alpha (TNF-α, interleukin (IL)-1 β, and IL-6) not only in the periphery but also in the brain [134–136] which causes neuroinflammation and brain damage [137, 138]. This offers an effective framework for examining the part played by peripheral inflammation in the onset and/or progression of neurodegenerative diseases. In this context, studies have demonstrated that rats given a subseptic dose of LPS (100 µg/kg, i.p.) and treated with capsaicin (1.5 mg/kg, i.v. or i.p.) had significantly higher brain GSH by 20.6% and 15.9%, respectively as well as markedly decreased nitric oxide in serum compared to controls [133]. Additionally, it has been shown that capsaicin-sensitive afferents running in vagus nerves are involved in maintenance of the brain’s redox status under basal as well as systemic inflammatory conditions. Bilateral subdiaphragmatic vagotomy or defunctionalization of capsaicin-sensitive vagal afferents by perivagal capsaicin (1%) application had a significant effect on brain oxidative stress, enhancing lipid peroxidation and lowering GSH levels in saline and LPS (300 µg/kg, i.p.)-treated rats. According to the study’s findings, vagal capsaicin-sensitive sensory afferents play a role in the upkeep of the brain’s redox state [139].

Toll-like receptor 4 (TLR4) present on macrophages, monocytes, dendritic cells and other immune cells is activated by LPS, causing the release of oxygen free radicals, monocyte chemoattractant protein-1 (MCP-1), and potent pro-inflammatory cytokines such as TNF-α and IL-1β which signal to the brain *via* the vagal fibers [140, 141]. Several in vitro studies have suggested that capsaicin was able to inhibit the release of oxygen free radicals and inflammatory mediators from peritoneal macrophages. Thus, pre-incubating rat peritoneal macrophages with 10 µM capsaicin has been shown to inhibit the production of superoxide radical (O_2_**˙ˉ**), hydrogen peroxide (H_2_O_2_), and nitrite. Piperine, another TRPV1 agonist, also had these effects, albeit at much greater concentrations (500 µM). Additionally, peripheral macrophages from capsaicin-fed rats (5 mg/kg/day) for 2 weeks similarly produced less oxygen free radicals compared to the macrophages from the control groups [142] as well as less prostaglandin E2, leukotrienes B4 and C4 but increased the release of 6-keto PGF1α [143]. Curcumin (turmeric) shared the above effects with capsaicin [142, 143], thereby, suggesting an antioxidative mechanism being involved. Another study by Chen et al. [144] have demonstrated that capsaicin and its analogue resiniferatoxin both suppressed LPS and INF-γ-mediated expression of inducible NOS (iNOS) protein and mRNA and nitric oxide production in RAW264.7 macrophages (IC50: 10 µM). The vanilloids also inhibited the LPS-induced stimulation of nuclear factor kappa-B (NF-κB) and activator protein 1. Capsaicin but not resiniferatoxin, prevented the LPS-induced expression of cyclooxygenase type 2 (COX-2) and the synthesis of prostaglandin E2 (PGE2). Capsaicin’s effects may involve both TRPV1-dependent and -independent mechanisms since capsazepine, imitated rather than reversed these effects of capsaicin.

Capsaicin thus may act in the periphery to reduce oxidative and inflammatory signalling to the brain *via* the vagal sensory fibers expressing TRPV1 receptors which are involved in immune-to-brain signalling [145, 146]. Stimulation of the vagus nerve has been shown to decrease brain levels of pro-inflammatory cytokines, activated microglia and macrophages during sepsis, resulting in turn in decreased neuroinflammation [147, 148].

Capsaicin can also act to inhibit the release of reactive oxygen radicals and mediators of inflammation from activated microglia. In their study, Zheng et al. [149] have shown that capsaicin (50 or 100 µg/ml) inhibited nitric oxide, INF-α, IL-1β and IL-6 production by murine BV-2 microglia cells exposed to LPS in vitro. Capsaicin also inhibited protein expression of COX-2 and iNOS, IκB phosporylation and NF-κB translocation into the nucleus of BV-2 cells. The results of this investigation in addition indicated that capsaicin at 25–500 µg/ml had no discernible impact on cell viability in the MTT cytotoxicity assay.

Studies that employed genetic TRPV1 channel deletion or desensitization have shown that TRPV1 channel receptors mediate protective function during sepsis. Thus, Guptill et al. [150] have demonstrated increased mortality risk from sepsis caused by caecal ligation and puncture in TRPV1 null mice or following desensitization of TRPV1 channel with intrathecal resiniferatoxin. Capsazepine 50 µg s.c., on the other hand, raised the risk of mortality. TRPV1 deletion or desensitization was associated with increased blood bacterial count and serum nitric oxide compared to controls. In a different investigation, LPS (10 mg/kg, i.p.) was used to produce sepsis. Compared with the wild-type controls, mice with genetically deleted TRPV1 channels had higher amounts of TNF-α and nitric oxide in peritoneal exudates (but not in plasma). Other researchers have found that sepsis induced in mice by caecal ligation and puncture was worsened by the ablation of TRPV1 channels. These mice exhibited further increases in plasma levels of the hepatocellular enzyme AST, creatinine, and lipase as compared to their wild-type counterparts. Additionally, they revealed a reduction in number of viable mononuclear cells, impaired phagocytic function, a decline in MCP-1, nitric oxide, and reactive oxygen species as well as an increase in the amounts of the cytokines TNF-α, IL-6 and IL-10 in peritoneal lavage. Moreover, bacterial clearance was less as compared to the wild-type mice with sepsis [151]. It is obvious from the above studies that TRPV1 protects against inflammation in sepsis, helps bacterial clearance and therefore postpones the establishment of a more severe systemic inflammatory state.

**Fig. 5:**
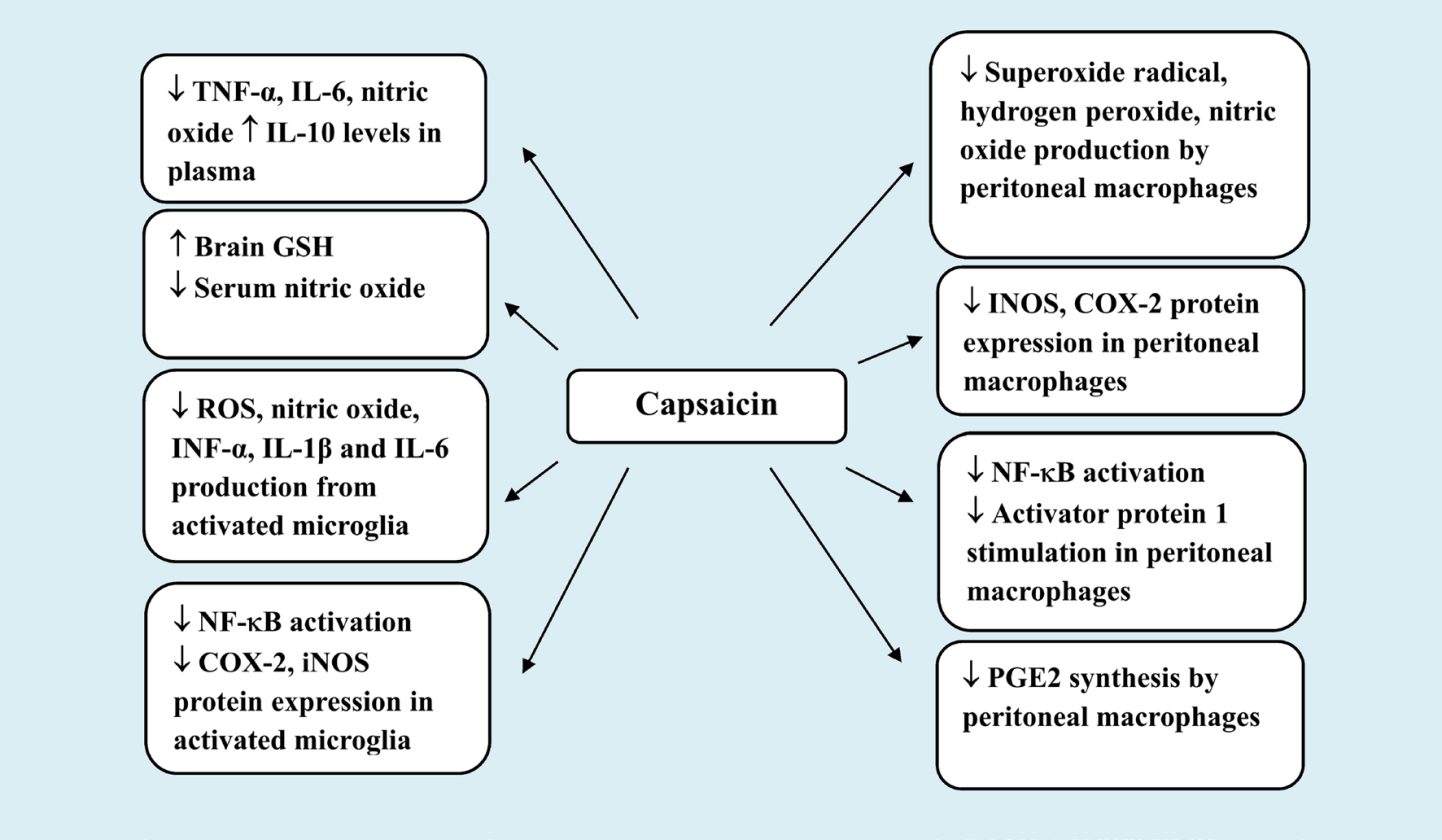
Effects of capsaicin in systemic inflammation/sepsis

Studies have shown that endotoxaemia causes a rise in CGRP release. Patients with sepsis exhibited significantly elevated plasma CGRP levels compared to their controls [152]. Moreover, Orliac et al. [153] have demonstrated increased density of CGRP-positive nerves and amplification of the anandamide-induced release of CGRP and vasorelaxation in mesenteric beds that followed i.p. administration LPS (5 mg/kg). These observations suggest that TRPV1 receptors are activated during endotoxaemia releasing CGRP from sensory nerve endings. This increase in CGRP may account for the vasodilatation and the decrease in vascular resistance in sepsis i.e., the hyperdynamic septic state [152]. It’s intriguing to think therefore that, the elevated levels of CGRP and resultant vasodilatation in sepsis could possibly represent a protective mechanism that enhances bacterial clearance (Fig. 5).

### Epilepsy

Neurobehavioral signs and symptoms of epileptic seizures are caused by a constellation of rapid, recurrent, excessively synchronized discharges in brain neurons. In epilepsy, brain excitatory and inhibitory synaptic functions are out of balance [154]. TRPV1 receptors are thought to play a role in the pathophysiology of epileptic seizures [155]. According to an in vivo research, treatment of mice with small doses of capsaicin systemically, prevented the development of seizures that were evoked by either kainic acid or pentylenetetrazole (PTZ). In this investigation, Lee et al. [156] used an i.p. injection of the glutamate analogue kainic acid to produce epilepsy in mice. Either 0.33 or 1 mg/kg of capsaicin was subsequently administered subcutaneously. Capsaicin lowered the seizure score and electrical seizure activity in the parietal cortex in a dose-dependent manner. Kainic acid-induced oxidative stress evidenced by increased levels of thiobarbituric acid reactive compounds in brain tissue, which imply an increase in lipid peroxidation, decreased antioxidant capacity, and increased H_2_O_2_ in blood. Additionally, there were elevated levels of TNF-α and IL-6 in the brain, a rise in body temperature, and apoptotic cell death in the hippocampal region. Capsaicin therapy slowed these alterations down.

**Fig. 6:**
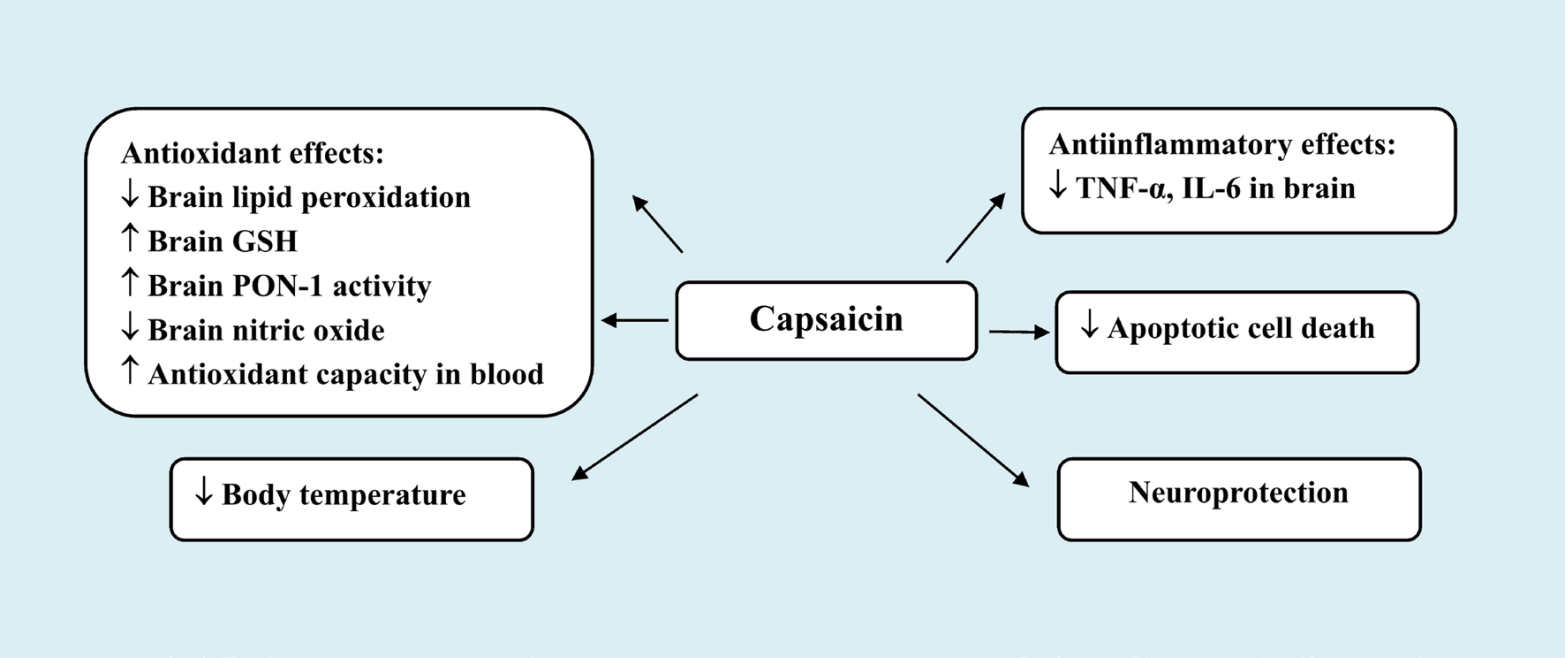
Effects of small dose systemic capsaicin in experimental epilepsy

Other experiments have shown that the mean seizure score in status epilepticus elicited in the rat by repeated i.p. doses of PTZ decreased when capsaicin was administered at 1 or 2 mg/kg [157]. The latter, a non-competitive antagonist of the GABA-A receptor, is frequently employed to simulate human epilepsy [158]. In the brains of PTZ-treated rats, capsaicin demonstrated antioxidant properties that attenuated the rise in lipid peroxidation, nitric oxide and the decrease in reduced glutathione (GSH) and paraoxonase-1 activity. The study also demonstrated that PTZ therapy thinned the hippocampus and caused dark, flattened neurons. Additionally, the substantia nigra’s dopaminergic pigmented cells were smaller and fewer in number, and the cerebral cortex had a high number of acidophilic neurons. Capsaicin and phenytoin both inhibited these neurodegenerative alterations, and the combination of the two virtually totally prevented them [157]. The above data clearly indicated a potential anti-seizure and a neuroprotective action for systemically administered small doses of capsaicin as a result of lowering the levels of oxidative stress and neuroinflammation. The contribution of TRPV1 activation in the capsaicin’s effect, was, however, not determined and awaits further studies (Table 1) (Fig. 6).

**Table 1:** Summary of the possible pathways underlying the neuroprotective effects of capsaicin/hot pepper

↓ Oxidative stress (↓ MDA, ↓ NO, ↑ GSH) [72, 132, 155, 156].
↓ Excitotoxicity [73, 90].
↓ Release of pro-inflammatory cytokines (↓ TNF-α and IL-6) → inhibits neuroinflammation [91].
↓ Activated microglia and macrophages → ↓ Expression of COX-2, iNOS, IκB phosporylation and NF-κB translocation → inhibits neuroinflammation [72, 143, 148].
↑ Release of vasocative neuropeptides e.g., CGRP, somatostatin, and SP → protective vasodilatation [75, 79].
↓ Caspase-3 activation [74, 84].
Changes in release of synaptic neurotransmitters [14, 79].
↓ Neuronal activity [15].
↑ Stimulation of abdominal vagal afferent fibers expressing TRPV1 nociceptors → signals to brain → inhibits oxidative stress [138].
Effect on thermoregulation (↓ body temperature) → neuroprotection [70].
↓ 5-lipoxygenase → ↓ leukotrienes → inhibits neuroinflammation [109, 110],
↑ Somatostatin in circulation → modulates inflammation (sensocrine function of capsaicin-sensitive nociceptors) [24, 25],
↓ Blood coagulation and platelet aggregation (also DHC) [80].
↓ Oxidation of LDL [84].

*MDA* malondialdehyde; *GSH* reduced glutathione, *NO* nitric oxide,* IL*-1β interleukin-1beta, IL-6 interleukin-6, *TNF*-α tumour necrosis factor- α, *CGRP* calcitonin gene-related peptide, *SP* substance P,* iNOS* nitric oxide synthase, *NF*-κB nuclear factor kappa B, *COX*-2 cyclooxygenase-2

## Conclusion

Capsaicin has long been used as a probe to explore sensory nerve-mediated mechanisms. The TRPV1 receptor’s molecular site of action was discovered, and the expression of this receptor in neurons and non-neuronal cell types in the brain suggested that it is involved in a variety of physiologic processes in the brain. A growing body of research suggests that the TRPV1 channel receptor may be a target for the treatment of a variety of pathologic brain conditions. Capsaicin, a TRPV1 agonist found in hot peppers, lowers brain oxidative stress and neuroinflammation. This property may help to prevent neuronal death in diseases like Parkinson’s or Alzheimer’s, cerebral stroke, epilepsy, and systemic inflammatory disease.

## Data Availability

All data supporting the findings of this study are available within the paper.
